# Biomarkers of immunothrombosis and polymorphisms of *IL2*, *IL6*, and *IL10* genes as predictors of the severity of COVID-19 in a Kazakh population

**DOI:** 10.1371/journal.pone.0288139

**Published:** 2023-06-30

**Authors:** Assiya Yessenbayeva, Bakytbek Apsalikov, Meruyert Massabayeva, Maksut Kazymov, Aizhan Shakhanova, Zhanna Mussazhanova, Irina Kadyrova, Nurlan Aukenov, Nurlan Shaimardanov

**Affiliations:** 1 Medical University Semey, Semey, Republic of Kazakhstan; 2 Department of Tumor and Diagnostic Pathology, Nagasaki University, Nagasaki, Japan; 3 High Medical School, Faculty of Medicine and Health Care, Al Farabi Kazakh National University, Almaty, Republic of Kazakhstan; 4 Shared Resource Laboratory of Karaganda Medical University, Karaganda, Republic of Kazakhstan; 5 Department of Health and Human Resources, Ministry of Health of the Republic of Kazakhstan, Astana, Republic of Kazakhstan; University of Salamanca/University Hospital of Salamanca, SPAIN

## Abstract

**Objectives:**

To study the role of biological markers of immunothrombosis and polymorphisms of cytokine genes *IL2*, *IL6*, *IL10* and their influence on the severity of COVID-19 in a Kazakh population.

**Methods:**

A total of 301 patients of Kazakh nationality with a confirmed diagnosis of COVID-19 participated in the retrospective study, including 142 patients with severe and 159 with a mild course. Single nucleotide polymorphisms *IL2R* rs1801274, *IL6* rs2069840, and *IL10* rs1800872 were genotyped by real-time PCR. Activated partial thromboplastin time, normalized ratio, prothrombin index, prothrombin time, fibrinogen prothrombin time, fibrinogen, D-dimer, and C-reactive protein analysis were also conducted.

**Results:**

The average age of patients with severe COVID-19 is higher than of patients with mild COVID-19 (*p* = 0.03). The findings showed that fibrinogen, D-dimer, and C-reactive protein were significantly greater in the group of patients with severe COVID-19 (*p* = 0.0001). A very strong correlation between the severity of COVID-19 with the D-dimer and C-reactive protein (*p* = 0.9) (*p* = 0.02) was found.

**Conclusion:**

The results of our study confirm that D-dimer, fibrinogen, and CRP are biomarkers of inflammation and hypercoagulation that serve as predictors of immunothrombosis affecting the severity of COVID-19. D-dimer is also associated with *IL10* rs1800872 gene polymorphism in the Kazakh population with severe COVID-19.

## Introduction

Since the beginning of the pandemic declared by the World Health Organization in 2020, coronavirus has remained a public health burden globally over. Currently, researchers face the challenge of finding new biomarkers for the progression of severe acute respiratory syndrome coronavirus 2 (SARS-CoV-2) caused by a coronavirus discovered in 2019 (COVID-19) [1]. Understanding the process of disease progression from asymptomatic to extremely severe is closely related to understanding the mechanisms underlying the pathogenesis of cell damage and the immune response. It is known that some of the infected subjects have no symptoms or the symptoms are mild, whereas in others the disease is severe and even fatal [2]. The severity of COVID-19 depends on a number of factors, among which metabolic syndromes such as diabetes mellitus, arterial hypertension, and obesity can be distinguished; the age of patients and the presence of some comorbidities can also be attributed to predictors of severity [3, 4]. The overall situation has significantly improved and the disease has become more controlled with the advent of vaccines and widespread vaccination [5]. Nevertheless, there are still severe cases of the disease, and questions about the duration of the immune response and the weakening of immunity remain [6], which creates prerequisites for further study of the pathogenesis of the severity of COVID-19.

Our study is aimed to find biological markers that are clinically useful for predicting the severity of COVID-19 and determining immunothrombosis; we suggested that polymorphisms of cytokine genes *IL2*, *IL6*, and *IL10* might play a role in the clinical manifestation and outcome of COVID-19 infection and might influence the production of the biological markers.

## Methods

### Subjects

A total of 301 patients of Kazakh nationality with a confirmed diagnosis of COVID-19 participated in the retrospective case-control study from April to June 2022. The criteria for inclusion in the study were a confirmed diagnosis of COVID-19 (a positive result of a PCR test for virus RNA SARS-CoV-2) unvaccinated, and age over 18 years. Among the total number of patients, 142 had severe COVID-19 with more than 50% lung damage, SpO2 <90, respiratory rate > 30/min, and were on inpatient treatment in intensive care unit hospitals in Semey, Kazakhstan. The control group included 159 people with mild symptoms, without lung damage and clinical symptoms of the disease, SpO2 > 95%, who were observed on an outpatient basis in Semey polyclinics. The severity of COVID-19 was determined in accordance with the criteria Clinical Protocol for the diagnosis and treatment of COVID-19 in adults No. 130 of April 1, 2021, approved by the Joint Commission on the Quality of Medical Services of the Ministry of Health of the Republic of Kazakhstan (S1 Table).

### Laboratory tests

Blood collection for the study was carried out upon admission to the hospital prior to anticoagulant therapy. Venous blood was collected in 3 ml tubes with 3.2% sodium citrate to study the parameters of blood coagulation function, and tests were performed on a Sysmex CS-2500 analyzer (Siemens Healthineers). The C-reactive protein was determined using a Cobas 8000 analyzer (Roche Diagnostics GmbH). All tests were carried out using commercial kits in accordance with the instructions of the manufacturer. Reference range indicators are: activated partial thromboplastin time (aPTT)– 25–35 s, international normalized ratio (INR)– 0.8–1.1, prothrombin index (PTI)– 80–110%, prothrombin time (PT)– 9–15 s, fibrinogen (Fgn)– 1.8–4 g/L, D-dimer– 220–500 ng/mL, and C-reactive protein (CRP) < 10 mg/L.

DNA isolation was performed using QIAamp DNA MiniKit kits (QIAGEN, Germany), and DNA concentration was measured using NanoDrop 1000 (Thermo Scientific, Waltham, MA, USA). The DNA was frozen and stored at -20°C. Genotyping was performed with CFX96 ™ Real-Time PCR (Bio-Rad) using primers and TaqMan probes. 40 ng of genomic DNA and 20 μl of reagent TaqMan Genotyping MasterMix in 96-well plates (reagents produced by Life Technologies). Sequences of used primers IL10 (rs1800872) forward 5′- GGTGAGCACTACCTGACTAGC-3′ and reverse 5′-AAAGCCACAATCAAGGTTTCCC-3′; IL6 (rs2069840) forward 5′ -ACGTTGGATGCCAGGCAGCAACAAAAAGTG-3′ and reverse 5′ -ACGTTGGATGCTGTCCAAGAATAAACTGCC-3′; IL2R (rs1801274) forward 5′- ACGTTGGATGCTTCCAGAATGGAAAATCCC-3′ and reverse 5′- ACGTTGGATGCTGTGACTGTGGTTTGCTTG-3′. The amplification program included pre-denaturation at 95°C for 10 minutes, followed by 50 cycles of 92°C for 15 seconds and 62°C for 1 minute.

### Ethical approval statement

This study was approved by the Ethics Committee of Semey Medical University, Semey, Kazakhstan, Protocol #2 of October 28, 2020 and meets the requirements of the Helsinki Declaration, the World Medical Association. Informed written consent was obtained from all participants of the study prior to the participation in the study, all personal data were confidential.

### Statistical analyses

Statistical analysis was performed using SPSS version 20 (IBM Corp.) and SNPStat version 2.2.1. All variables were checked for distribution normality using Shapiro–Wilk test. To test for the normality of the sample distribution, we used the Kolmogorov-Smirnov test, since the sample differed from the norm, we used the U-Mann Whitney test for two independent samples. Cross-tabulation was used to identify the distribution of risk factors and outcomes to analyze risk factors association; the odds ratio (OR) indicator with a 95% confidence interval (CI) was calculated. To study the risk factors association, multiple logistic regression was performed. Logistic regression in co-dominant, dominant, and recessive models was used to study the difference between phenotypic variations and genotypes. P < 0.05 was considered to indicate a statistically significant difference for a single test. To assess the correspondence of the genotype frequency distributions according to the Hardy-Weinberg equilibrium (HWE) the χ2 test was used (p > 0.05 was estimated as compliance with the Hardy–Weinberg equilibrium).

## Results

A total of 301 patients with a confirmed diagnosis of COVID-19 were included in the study. Among them, 142 of these patients had severe COVID-19 with more than 50% lung damage and 159 people had mild symptoms with no lung damage. The severity of COVID-19 did not differ by gender (*p* = 0.573) (Table 1). The median age of patients with severe and mild disease in each age group (18–29 years; 30–39; 40–49; 50–59 and 60 years and over) was compared and no statistically significant difference was found between them (Table 1).

**Table 1:** Clinical characteristics of COVID-19 patients.

	Severe (n = 142)	Mild (n = 159)	P value
Gender			
Male, n (%)	70 (48.95)	83 (52.2)	0.573
Female, n (%)	73 (51.05)	76 (47.8)	
Age, Ме (Q1-Q3)	39 (29–51)	33 (25–43)	0.03
Age categories, n (%)			
18–29	37 (26.06)	31 (19.5)	0.091
30–39	39 (27.46)	36 (22.64)	0.175
40–49	28 (19.72)	29 (18.24)	0.955
50–59	29 (20.42)	35 (22.01)	0.368
60 and older	9 (6.34)	28 (17.61)	0.255
	Ме (Q1-Q3)		
aPTT, s	27.8 (25.6–29.62)	26.5 (23.6–29.8)	0.318
INR	1.06 (0.97–1.13)	1.06 (0.96–1.13)	0.494
PTI, %	78.25 (71.6–87.42)	88.65 (83.65–95.26)	0.001
PT, s	12.5 (11.6–13.4)	12.8 (12.03–14.12)	0.012
Fgn, g/L	4.3 (3.8–5.1)	3.0 (2.2–3.3)	0.0001
CRP, mg/L	46.85 (23.27–62.62)	0.9 (0.4–1.1)	0.0001
D-dimer, ng/mL	170 (690–4500)	150 (105–198)	0.0001

*Note*. All correlations are significant at *p* < 0.05 *Abbreviations*: n = number, aPTT = activated partial thromboplastin time, INR = international normalized ratio, PTI = prothrombin index, PT = prothrombin time, CRP = C-reactive protein, Fgn = fibrinogen

Table 1 shows the statistically significant differences between the compared groups using the Mann-Whitney test. The mean values of PTI (*p* = 0.001), PT (*p* = 0.012), fibrinogen (*p* = 0.0001), D-dimer (*p* = 0.0001) were significantly higher in the group of patients with severe COVID-19. Very high values of C-reactive protein were more common in the group of patients with severe COVID-19 (*p* = 0.0001).

C-reactive protein and D-dimer are significantly increased in people with severe disease, there is also a slight increase in aPTT and a decrease in PTI and PV in patients with severe COVID-19. The chance of developing high D-dimer values in patients with severe COVID-19 was 4.18 times higher compared to patients with mild COVID-19, which increased thrombotic complications in patients with severe COVID-19. The chance of developing high values of C-reactive protein was 2.33 times higher in the group of patients with severe COVID-19 compared with patients with mild COVID-19.

A very strong relationship (rS = 0.9) (*p* = 0.02) was found when studying the correlation between the severity of COVID-19 and D-dimer. There is also a very strong correlation between the C-reactive protein and the severity of COVID-19 (rS = 0.9) (*p* = 0.02).

Allelic and genotypic analysis of polymorphisms of the studied cytokine genes showed an association between *IL10* rs1800872 and the severity of COVID-19 (Table 2). However, this result does not take into account the correction for multiple comparisons (*p* ≤ 0.005).The association of the *IL2R* rs1801274 and *IL6* rs2069840 gene polymorphisms with the severity of COVID-19 was not found (S2 Table). No deviations of the Hardy–Weinberg equilibrium (HWE) were found in the distribution of genotype frequencies for each SNP (*p* > 0.2).

**Table 2:** Association of *IL10* rs1800872 gene polymorphisms with the severity of COVID-19.

SNPs	Severe	Mild	OR (95% CI)	χ^2^	P value
*IL10*					
(rs1800872)	0.511	0.594	0.71(0.52–0.98)	4.24	0.04
G	0.489	0.406	1.40 (1.02–1.94)		
T					
G/G	0.227	0.327	0.60 (0.36–1.01)		
G/T	0.567	0.535	1.14 (0.72–1.80)	4.78	0.03
T/T	0.206	0.138	1.61(0.88–2.96)		

*Note*. SNP: single nucleotide polymorphism

We performed an associative analysis of the genotypes studied by SNP with D-dimer. We found that *IL10* rs1800872 was significantly associated with an increase in D-dimer level in the dominant, codominant, and Log-additive genetic models (*p* = 0.008) (Table 3).

**Table 3:** Association of polymorphisms of *IL10* rs1800872 gene with D-dimer response and severity of COVID-19.

Model	Genotype	D-dimer	D-dimer	OR (95% CI)	P value	AIC	BIC
		Severe	Mild				
Co	G/G	54 (34.2%)	30 (21.3%)	1.00	0.026	412.3	423.4
	T/G	83 (52.5%)	82 (58.2%)	1.78 (1.04–3.05)			
	T/T	21 (13.3%)	29 (20.6%)	2.49 (1.21–5.09)			
Do	G/G	54 (34.2%)	30 (21.3%)	1.00	0.013	411.3	418.7
	T/G-T/T	104 (65.8%)	111 (78.7%)	1.92 (1.14–3.23)			
Re	G/G-T/G	137 (86.7%)	112 (79.4%)	1.00	0.092	414.7	422.1
	T/T	21 (13.3%)	29 (20.6%)	1.69 (0.91–3.12)			
Ov	G/G-T/T	75 (47.5%)	59 (41.8%)	1.00	0.33	416.6	424
	T/G	83 (52.5%)	82 (58.2%)	1.26 (0.79–1.98)			
Log-additive	---	---	---	1.60 (1.13–2.28)	0.008	410.5	417.9

*Note*: n = number, Co = codominant, Do = dominant, Re = recessive, Ov = over-dominant, OR = odds ratio, CI = confidence interval

The results indicated that there is no relationship between the D-dimer and the polymorphisms of the *IL2R* rs1801274 and *IL6* rs2069840 genes (S3 Table). An associative analysis of polymorphisms of *IL2R* rs1801274, *IL6* rs2069840, and *IL10* rs1800872 genes with aPTT, INR, PTI, PT, Fgn, and CRP blood parameters was also performed, but no significant associations were found (p > 0.05) (data not shown).

## Discussion

The pathophysiology associated with severe COVID-19 reflects a complex interaction between massive inflammatory activation in response to infection, inappropriate host immune responses, and as a consequence hypercoagulation and immunothrombosis [7]. The basis of immunothrombosis or otherwise thromboinflammation is the inhibition of coagulation, the formation of procoagulant mediators, and the activation of platelets and vascular endothelium [8].

Non-specific biomarkers of immunothrombosis include certain pro and anti-inflammatory cytokines, such as interleukin-2 (IL-2), interleukin-6 (IL-6), and interleukin-10 (IL-10), as well as C-reactive protein (CRP), D-dimer, fibrinogen (Fgn), prothrombin time (PT), prothrombin index (PTI), and activated partial thromboplastin time (aPTT) [9, 10]. CRP has been found to be an early indicator of the severity of COVID-19 [9]. A meta-analysis conducted in 2020 showed that an increase in CRP levels correlates with the level of inflammation and the severity of COVID-19 disease and increases the risk of adverse outcomes by 4 times [11]. Our study also demonstrates significant differences in CRP in the severity of COVID-19. A previous study also showed that ESR and CRP are biomarkers of inflammation and were significantly elevated in patients with COVID-19 [12].

There have been numerous studies on the blood coagulation system in COVID-19 showing that the severity of the course is based on dysregulation of the immune system and hypercoagulation along with inflammatory processes which lead to immunothrombosis. D-dimer and fibrinogen are the main markers of hypercoagulation and thrombotic load. There are a number of contradictory data on the effect of D-dimer on the severity of COVID-19 [13]. Reported an increase in D-dimer and fibrinogen levels in mild disease, whereas [14] meta-analysis demonstrated high levels of D-dimer, indicating hypercoagulation, which predict the severity of the disease. In our study no relationship was found between the severity of COVID-19 and such indicators of the blood coagulation system as aPTT, INR, PTI and PT. However, our results confirm the important role of D-dimer and fibrinogen as biomarkers of the severity of COVID-19. We found a very strong correlation between the severity of the COVID-19 with the D-dimer and the C-reactive protein. It should be emphasized that we have found a connection between the polymorphism of the IL10 rs1800872 gene and the D-dimer.

The current study also assessed the relationship of polymorphisms of the cytokine genes *IL2*, *IL6*, and *IL10* with the severity of COVID-19 in Kazakh population. The results indicates no association of *IL2R* rs1801274, *IL6* rs2069840, *IL10* rs1800872 gene polymorphisms with the severe course of the disease. These results were unexpected, given the pathogenetic association of the severity of COVID-19 with a massive immune inflammatory response and the impact of the cytokine storm on the outcome of the disease. [15] state that single nucleotide polymorphisms play an important role in the antiviral reactions of the host, in resistance and predisposition to diseases and the severity of their course. Growing evidence suggests that some SNPs can influence the susceptibility and severity of COVID-19.[16] confirmed the role of the IL-6 cytokine and the genetic predisposition of the *IL6* gene variant in response to infection as predictors of the severity of COVID-19 and adverse outcomes. A meta-analysis conducted in 2022 on the study of the effect of *IL10* on sepsis and some inflammatory pathologies confirmed the relationship between the rs1800871, rs1800872, rs1800896 polymorphisms of the *IL10* gene and sepsis, and also suggested their role in inflammatory pathologies [17]. As a result of the study, it was possible to establish a significant association of rs1800871 and rs1800896 with sepsis in Asian populations/ It was reported that IL-10 is vital in the inflammatory response of the body to COVID-19 infection and directly correlates with the severity of the disease [18]. Abbood et al. (2023) studied the association of IL10 rs1800871, rs1800872, and rs1800896 polymorphism with mortality from various variants of COVID-19 in the Iranian population [19]. The obtained results indicated that the CC IL10 rs1800871, TT, and GG genotypes with rs1800872 and rs1800896 polymorphisms were significantly associated with mortality from COVID-19 compared to other genotypes [19]. Our results also confirm the association of the *IL10* rs1800872 polymorphism and D-dimer with the severity of COVID-19 in the Kazakh population. The results of the association of the *IL10* rs1800872 polymorphism of the TT genotype in the Kazakh population (P <0.02, OR 2.49, 95% CI 1.21–5.09) are consistent with the results obtained in the Iranian population (P <0.0001, OR 3.39, 95% CI 2.65–4.35) [19]. There is also a study of the Mexican population, where the effect of *IL10* genetic polymorphisms rs1800871 and rs1800872 on the clinical outcomes of COVID-19 has not been confirmed [20].

Тhus, it should be noted that this study complements the current knowledge about the association of IL10 rs1800872 gene polymorphism with the biomarker of immunothrombosis and the severity of COVID-19. In addition, it is the first study to search for this association in the Kazakh population. Our study also has some limitations such as a relatively small sample size, lack of data on COVID-19 variants, and no repeat testing for D-dimer, CRP, and coagulation in patients with mild disease. Nevertheless, the results may be of interest for sthe future studies on various ethnic groups.

## Conclusions

Our study demonstrates a probable association of IL10 rs1800872 gene polymorphism with the severity of COVID-19, as well as a strong relationship between IL10 rs1800872 gene polymorphism and D-dimer in Kazakh populations with severe COVID-19. D-dimer, CRP and fibrinogen are also associated with the severity of COVID-19.

## Supporting information

S1 Table COVID-19 severity criteria. (DOCX)

S2 Table Association of IL6 rs2069840, IL2R rs1801274 gene polymorphisms with the severity of COVID-19. (DOCX)

S3 Table Association of polymorphisms of IL6 rs2069840, and IL2R rs1801274 genes with D-dimer response and severity of COVID-19. (DOCX)

S4 Table STROBE statement—Checklist of items that should be included in reports of observational studies. (DOCX)
