# Supplementary material for: Biomarkers of immunothrombosis and polymorphisms of IL2, IL6, and IL10 genes as predictors of the severity of COVID-19 in a Kazakh population
Source: PLoS One. 2023 Jun 30;18(6):e0288139. doi: 10.1371/journal.pone.0288139 (PMC10313014; doi:10.1371/journal.pone.0288139)
Supplement: S1 Table — (DOCX) [file pone.0288139.s001.docx]

**Table SI. COVID-19 Severity Criteria**

| COVID-19  Severity Criteria | Mild | Moderate | Severe | Critical |
| --- | --- | --- | --- | --- |
| Body temperature | normal or  subfebrile, febrile | fever (often febrile) | fever (subfebrile, febrile less often - normal) | fever (subfebrile, febrile less often - normal) |
| Dyspnea/  shortness of breath | no shortness of breath | shortness of breath on exertion / minor shortness of breath | shortness of breath on slight exertion, talking, at rest/ moderate shortness of breath | severe shortness of breath/  shortness of breath at rest |
| Respiratory rate at rest | <20 per minute | 20-22 per minute | 23-30 per minute | >30 per minute |
| SpO2 | >95% | 94-95% | 90-93% | <90% |
| Chest X-ray/CT Scan (if available)  (the clinical picture does not always match the clinic) | no change | 1. Opacities in the lungs on X-ray, often of rounded morphology, with peripheral and lower distribution over the lungs;  2. CT 1-2 lung damage volume up to 50%. | 1. X-ray - signs of bilateral viral lung damage  2. CT 3-4 lung damage volume >50% | 1. X-ray - signs of significant bilateral viral damage to the lungs  2. CT -4 lung damage volume 75-100% |
| Heart rate  *(correlate with body temperature)* | 60-80 bpm | 80-100 bpm | 100-120 bpm | more than 120 bpm  Probable rhythm and conduction disturbances |
