# Supplementary material for: Biomarkers of immunothrombosis and polymorphisms of IL2, IL6, and IL10 genes as predictors of the severity of COVID-19 in a Kazakh population
Source: PLoS One. 2023 Jun 30;18(6):e0288139. doi: 10.1371/journal.pone.0288139 (PMC10313014; doi:10.1371/journal.pone.0288139)
Supplement: S2 Table — (DOCX) [file pone.0288139.s002.docx]

**Table SII. Association of *IL6* rs2069840, *IL2R* rs1801274 gene polymorphisms with the severity of COVID-19**

| SNPs | Severe | Mild | OR (95% CI) | χ^2^ | P value |
| --- | --- | --- | --- | --- | --- |
| *IL6* (rs2069840)  *C*  *G* | 0.303  0.697 | 0.288  0.713 | 1.08 (0.76 – 1.53)  0.93 (0.65 – 1.32) | 0.17 | 0.68 |
| *C/C*  *C/G*  *G/G* | 0.113  0.380  0.507 | 0.106  0.363  0.531 | 1.07 (0.52 – 2.20)  1.08 (0.68 – 1.72)  0.91 (0.58 – 1.43) | 0.15 | 0.7 |
| *IL2R* (rs1801274)  *А*  *G* | 0.331  0.669 | 0.365  0.635 | 0.86 (0.61 – 1.21)  1.16 (0.83 – 1.63) | 0.73 | 0.39 |
| *А/А*  *А/G*  *G/G* | 0.108  0.446  0.446 | 0.148  0.432  0.419 | 0.69 (0.35 – 1.39)  1.06 (0.67 – 1.68)  1.11 (0.70 – 1.77) | 0.70 | 0.4 |
| *Note.* SNP: single nucleotide polymorphism | | | | | |
