# Supplementary material for: Biomarkers of immunothrombosis and polymorphisms of IL2, IL6, and IL10 genes as predictors of the severity of COVID-19 in a Kazakh population
Source: PLoS One. 2023 Jun 30;18(6):e0288139. doi: 10.1371/journal.pone.0288139 (PMC10313014; doi:10.1371/journal.pone.0288139)
Supplement: S3 Table — (DOCX) [file pone.0288139.s003.docx]

**Table SIII. Association of polymorphisms of *IL*6 rs2069840, and *IL*2R rs1801274 genes with D-dimer response and severity of COVID-19**

| Model | Genotype | D-dimer  Severe | D-dimer  Mild | OR (95% CI) | P value | AIC | BIC |
| --- | --- | --- | --- | --- | --- | --- | --- |
| Association of *IL6* rs2069840 with D-dimer response with the severity of disease (n=301) | | | | | | | |
| Co | G/G  C/G  C/C | 87 (54.7%)  56 (35.2%)  16 (10.1%) | 69 (48.6%)  56 (39.4%)  17 (12%) | 1.00  1.26 (0.77-2.05)  1.34 (0.63-2.84) | 0.56 | 421.2 | 432.3 |
| Do | G/G  C/G-C/C | 87 (54.7%)  72 (45.3%) | 69 (48.6%)  73 (51.4%) | 1.00  1.28 (0.81-2.01) | 0.29 | 419.2 | 426.6 |
| Re | G/G-C/G  C/C | 143 (89.9%)  16 (10.1%) | 125 (88%)  17 (12%) | 1.00  1.22 (0.59-2.51) | 0.6 | 420 | 427.4 |
| Ov | G/G-C/C  C/G | 103 (64.8%)  56 (35.2%) | 86 (60.6%)  56 (39.4%) | 1.00  1.20 (0.75-1.91) | 0.45 | 419.7 | 427.2 |
| Log-additive | --- | --- | --- | 1.19 (0.85-1.66) | 0.31 | 419.3 | 426.7 |
| Association of *IL2R* rs1801274 with D-dimer response with the severity of disease (n=293) | | | | | | | |
| Co | G/G  A/G  A/A | 67 (43.2%)  63 (40.6%)  25 (16.1%) | 59 (42.8%)  66 (47.8%)  13 (9.4%) | 1.00  1.19 (0.73-1.94)  0.59 (0.28-1.26) | 0.18 | 407.8 | 418.8 |
| Do | G/G  A/G-A/A | 67 (43.2%)  88 (56.8%) | 59 (42.8%)  79 (57.2%) | 1.00  1.02 (0.64-1.62) | 0.94 | 409.2 | 416.6 |
| Re | G/G-A/G  A/A | 130 (83.9%)  25 (16.1%) | 125 (90.6%)  13 (9.4%) | 1.00  0.54 (0.26-1.10) | 0.085 | 406.2 | 413.6 |
| Ov | G/G-A/A  A/G | 92 (59.4%)  63 (40.6%) | 72 (52.2%)  66 (47.8%) | 1.00  1.34 (0.84-2.13) | 0.22 | 407.7 | 415 |
| Log-additive | --- | --- | --- | 0.88 (0.63-1.22) | 0.44 | 408.6 | 416 |
| *Note:* n = number, Co = codominant, Do = dominant, Re = recessive, Ov = over-dominant, OR = odds ratio, CI = confidence interval | | | | | | | |
